# Hysterectomy and thyroid cancer risk: A systematic review and meta-analysis

**DOI:** 10.1016/j.gloepi.2023.100122

**Published:** 2023-10-05

**Authors:** Roberto Fabiani, Patrizia Rosignoli, Irene Giacchetta, Manuela Chiavarini

**Affiliations:** a Department of Chemistry, Biology and Biotechnology, University of Perugia, Perugia 06122, Italy; b School of Specialization in Hygiene and Preventive Medicine, University of Perugia, Perugia 06132, Italy; c Department of Biomedical Sciences and Public Health, Section of Hygiene, Preventive Medicine and Public Health, Polytechnic University of the Marche Region, 60121 Ancona, Italy

## Abstract

**Background:**

Incidence rates of thyroid cancer have increased. Recent studies findings suggest that women who underwent a hysterectomy have an elevated relative risk of thyroid cancer. The aim of our meta-analysis is to summarize the evidence about the association between hysterectomy and thyroid cancer risk.

**Methods:**

PubMed, Web of Science, and Scopus database were searched for studies published up to 5 September 2023. The PRISMA statement was followed. Heterogeneity was explored with Q statistic and the I2 statistic. Publication bias was assessed with Begg's and Egger's tests.

**Results:**

Sixteen studies met the criteria. The pooled analysis showed a significantly 64% increment of thyroid cancer risk in association with any hysterectomy (OR 1.64, 95% CI 1.48–1.81; I2 = 28.68%, *p* = 0.156). Hysterectomy without oophorectomy was a stronger predictor of risk than hysterectomy with oophorectomy. The pooled analysis of data regarding hysterectomy without oophorectomy showed a statistically significant increment of thyroid cancer risk by 59%. Hysterectomy with oophorectomy was associated with an increase of thyroid cancer risk of 39% (OR 1.39, 95% CI 1.16–1.67; I2 = 42.10%, *p* = 0.049). Significant publication bias was not detected.

**Conclusions:**

Our findings help with decision making around these surgeries.

## Highlights

• This meta-analysis is the first to evaluate the association between hysterectomy and thyroid cancer risk.
• Risk of thyroid cancer was increased for women undergoing hysterectomy.
• the number of thyroid cancer asoociated with hysterectomy has important implications for public health guidance.

## Introduction

Thyroid cancer (TC) is the most common endocrine malignancy. Incidence rates of thyroid cancer have increased considerably during the last two decades. It is threefold as common in women as in men (10.1 per 100,000 women and 3.1 per 100,000 men). In both sexes, incidence rates were five times higher in high and very high Human Development Index countries than in low and medium Human Development Index countries [1]. Few established risk factors for thyroid cancer are known (such as radiotherapy treatment of the neck area, family history of thyroid cancer, some hereditary conditions, and excess body weight) [2,3]. There is no clear evidence of an association between reproductive or hormonal factors and thyroid cancer [4].

Hysterectomy is the most common gynaecologic surgery performed in women worldwide. Although it is the elective surgical procedure for treatment of uterine cancer, over 90% of procedures are associated to symptomatic benign gynaecological conditions (uterine fibroids, endometriosis, or unusual uterine bleeding) [5]. Often hysterectomy may also include the removal of both ovaries (bilateral salpinges-oophorectomy: BSO), a procedure that substantially reduce the risk of ovarian cancer [5]. The incidence rate of hysterectomy is decreasing over time in most countries, even though hysterectomies per 100,000 women swing among countries, in fact in 2018 it ranged from 12 in Denmark to 291 in Czech Republic [6]. Although considered safe, hysterectomy complications may occur which include infectious, venous thromboembolic and genitourinary and gastrointestinal tract injury [7]. Since both hysterectomy and BSO have the potential to induce evident changes in hormone levels [8], they can influence the risk of hormones related cancers. Discordant results regarding the association of hysterectomy-BSO with breast cancer risk were reported in several studies. In some cases, the breast cancer risk was lower after hysterectomy while no relation was observed in others [9]. Instead, in a previous meta-analysis an evident increment of risk in association with hysterectomy has been reported for both colorectal and kidney cancers [10,11]. Oophorectomy was also found to act as a risk factor for primary liver cancer [12].

Caini and colleagues have found that hysterectomy may play a role in the aetiology of thyroid cancer. Their review included four studies and didn't stratify hysterectomy by oophorectomy status [13].

Recently, several epidemiological studies have investigated the possible effect of hysterectomy, with or without BSO, on thyroid cancer risk with contrasting results.

Therefore, the present systematic review and meta-analysis was carried out to summarize and better understand the published epidemiologic evidence regarding the association between hysterectomy and thyroid cancer risk.

## Methods

We conducted this systematic review and meta-analysis according to the “preferred reporting items for systematic reviews and meta-analyses” (PRISMA) [14]. This research may be exempt from formal ethics review.

### Search strategy and study selection

A comprehensive literature search, without restrictions, was carried out until 5 September 2023 through PubMed, Web of Science, and Scopus databases to identify all the original articles on the association between hysterectomy and thyroid cancer risk. The following key words were used: hysterectomy AND (cancer OR tumour OR neoplasia OR “neoplastic disease” OR neoplasm) AND thyroid. In addition, to identify additional relevant publications, we manually examined the reference lists of included articles and recent relevant reviews. Study search results, initial duplication, search review and study selection were managed using Zotero (www.zotero.org).

### Inclusion and exclusion criteria

The inclusions criteria, organized on PICO model, were: was the association between hysterectomy and thyroid cancer in women. In particular:

- Population: Female Population;
- Intervention: any type of hysterectomy (with and without oophorectomy);
- Comparison: No hysterectomy;
- Outcomes: thyroid cancer, in particular, to search the databases, we used the following words: (cancer OR tumour OR neoplasia OR “neoplastic disease” OR neoplasm) AND thyroid;
- S: case–control, prospective, or cross-sectional study design;
- Finally, we included only articles, which reported a risk estimation (odds ratio, OR; relative risk, RR; or hazard ratio, HR) with 95% confidence intervals (CIs).

The exclusion criteria were:

- article not in English;
- some article type, such as case studies, commentaries and reviews;
- not reporting the association and/or the risk estimate between hysterectomy and thyroid cancer risk.

When there were several publications from the same study, the one with the largest sample was selected. Foreach potentially included study, two investigators independently conducted the selection, data abstraction, and quality assessment. Disagreements were resolved by discussion or in consultation with a third author. Although it is useful to have background information, reviews and meta-analyses were excluded. No studies were excluded based on weakness of design or data quality.

### Quality assessment

The study quality was assessed by a 9-star system, based on the Newcastle-Ottawa Scale (NOS) method [15]; the maximum score was 9 and a total score of ≥7 was used to indicate a high-quality study. To avoid selection bias, no study was excluded because of the quality criteria. Two authors individually performed the quality evaluation of each selected study and disagreements were settled by a joint re-evaluation with a third author.

### Data extraction

From the included studies, we extracted the following information: first author's last name, year of publication, country, study design and name, sample size (number of cases, controls, cohort size and incident cases), duration of follow-up for cohort studies, population characteristics (age, hysterectomy, reasons of surgery),whether the surgery included also unilateral/bilateral salpingo-ophorectomy (USO/BSO), age at surgery, risk estimates for the different categories of surgery (OR/RR/HR) with 95% CI sand adjustment of confounding factors. When multiple estimates were reported in the article, we abstracted those that adjusted for the most confounding factors. The outcome of interest in this study is thyroid cancer (TC).

### Statistical analysis

The association between hysterectomy and thyroid cancer risk in women was evaluated by version 3.0 of the ProMeta statistical program (IDoStatistics-Internovi, Cesena, Italy). For overall estimation, the relative risk and hazard ratio were taken as an approximation of the OR, and the meta-analysis was performed as if all types of ratio were ORs. We used the random effects model to calculate the summary OR and 95% confidence interval. Stratified analysis by study design (cohort and case-control studies), surgery categories (any hysterectomy, hysterectomy without oophorectomy, hysterectomy-BSO and hysterectomy-USO) and age at surgery (in the case of hysterectomy without oophorectomy) was also performed.

The chi-square-based Cochran's Q statistic and the I2 statistic were used to evaluate heterogeneity in results across studies [16]. The I2 statistic yields results ranged from 0%to 100% (I2 = 0–25%, no heterogeneity; I2 = 25–50%, moderate heterogeneity; I2 = 50–75%, large heterogeneity; and I2 = 75–100%, extreme heterogeneity) [17]. Results of the meta-analysis may be biased if the probability of publication is dependent on the study results. The methods of Begg and Mazumdar [18] and the methods of Egger et al. [19] were used to detect publication bias. Both methods were tested for funnel plot asymmetry. The former was based on the rank correlation between the effect estimates and their sampling variances, and the latter was based on a linear regression of a standard normal deviate on its precision. If a potential bias was detected, we further conducted a sensitivity analysis to assess the robustness of combined effect estimates, and the possible influence of the bias, and to have the bias corrected [18,19]. We also conducted a sensitivity analysis to investigate the influence of a single study on the overall risk estimate, by omitting one study in each turn. We considered the funnel plot to be asymmetrical, if the intercept of Egger's regression line deviated from zero, with a *p*-value <0.05.

## Results

### Studies selection

**Fig. 1:**
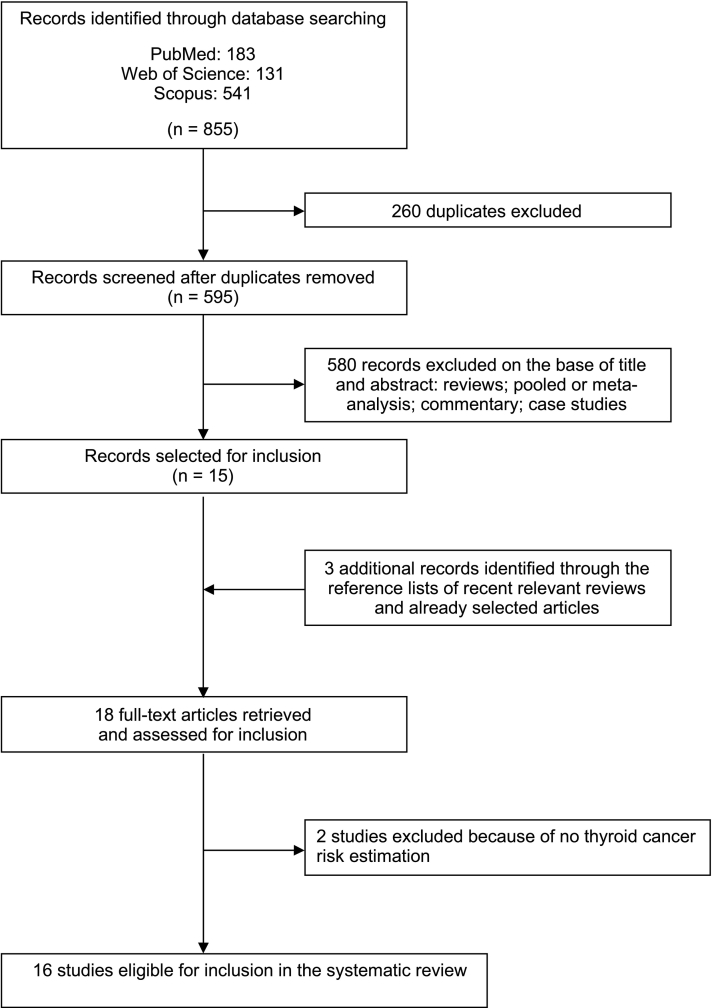
PRISMA flowchart of studies selection about the association between hysterectomy and risk of thyroid cancer.

From the primary literature search through PubMed (*n* = 183), Web of Science (*n* = 131) and Scopus (*n* = 541) databases, and after removing duplicates (*n* = 260), we identified 595 records for title and abstract revision (Fig. 1). Among these, 580 articles were excluded because they did not investigate the association between hysterectomy and thyroid cancer risk. Fifteen articles were subjected to full-text revision. Manual searching of reference lists of both selected articles and recent relevant reviews led to the identification of 3 additional items. Subsequently, 2 articles were excluded because they did not report the risk estimation. Therefore, at the end of the selection process, 16 studies were included in the systematic review and meta-analysis [[20], [21], [22], [23], [24], [25], [26], [27], [28], [29], [30], [31], [32], [33], [34], [35]].

### Study characteristics and quality assessment

Principal characteristics of the 16 selected studies evaluating the association between hysterectomy and thyroid cancer risk are reported in Table 1. They were published between 1997 and 2021. Five studies were conducted in USA [26,28,29,33,34]; two in Korea [20,23], Finland [32,35], Sweden [25,27] and Australia [21,22]; and one each in China [30], France [24] and New Caledonia [31]. Four were case-control studies [22,31,33,34] considering a total of 1680 cases and 2005 controls; 14 were cohort studies [20,21,[23], [24], [25], [26], [27], [28], [29], [30],^32^,^35^] considering a total population of 7,575,968of subjects and 20,697 incident thyroid cancer cases after exclusion of one study [25] which reported the same data on the same population of a previous publication [27]. In total 483,513 hysterectomised women were considered. Thirteen studies reported data for “any hysterectomy” not considering the concomitant oophorectomy [20,22,[24], [25], [26], [27], [28], [29], [30], [31], [32], [33], [34], [35]] while eight studies reported data for hysterectomy without oophorectomy [[21], [22], [23], [24],^26^,^31^,^33^,^34^]. Ten studies reported the thyroid cancer risk in association with both hysterectomy and bilateral salpingo-oophorectomy (BSO) [21,22,24,[26], [27], [28],[31], [32], [33], [34]] while four studies reported the thyroid cancer risk in association with both hysterectomy and unilateral salpingo-ophorectomy (USO) [21,31,32,34]. Seven out of 16 articles included in the systematic review reported the estimation of thyroid cancer risk as a function of age at surgery. [[21], [22], [23], [24], [25],^31^,^33^]

**Table 1:** Characteristics of studies on hysterectomy in association with thyroid cancer risk.

Author, year Location	Study design, Study name	Study population Subjects Cases/controls Follow-up Age Reason of surgery	Hysterectomy/ Oophorectomy (Assessment)	Age at surgery	OR/RR/HR/SIR (95% CI)	Matched or adjusted variables	QS^a^
Jin et al., 2021 Korea [20]	Prospective cohort Korean Genome and Epidemiology Study	Population: 107,365 Hysterectomy: 11,295 Cases: 1303 Follow-up: 12 y Age ≥ 40 y	No surgery Any hysterectomy (Self-reported)		Ref. 1.00 1.73 (1.48–2.01)	Age, BMI^b^, hypertension, diabetes mellitus, thyroid disease histories, occupation, smoking, alcohol, oophorectomy, number of children, use of oral contraceptive	9
Wilson et al., 2021 Australia [21]	Population-based retrospective cohort	Population: 838,237 Hysterectomy: 74,056 Cases: 2003 Follow-up: 27 y Age at entry: 29.9–39.6 y Benign indication	No surgery Hysterectomy without oopherectomy (Hospital record) Hysterectomy-USO4 Hysterectomy-BSO5	<45 y 45–54 y >55 y <45 y 45–54 y >55 y	Ref. 1.00 1.38 (1.19–1.60) 1.38 (1.18–2.53) 1.46 (1.16–1.83) 0.87 (0.48–1.56) 0.84 (0.44–1.64) 1.18 (0.90–1.54) 1.19 (0.78–1.80) 1.11 (0.76–1.63) 1.31 (0.73–2.35)	Age, parity, remoteness category, SEIFA^c^ quintile, fibroids, endometriosis, prolapse	9
Rahman et al., 2021 Australia [22]	Population-based case-control Queensland Thyroid Cancer Study (QTCS)	Cases: 685 Hysterectomy: 159 Age: 51 (40–60) y Control: 785 Hysterectomy: 126 Age: 52 (41–62) y Bleeding disorders, prolapse, cancer or other reason	No surgery Any hysterectomy Hysterectomy without oopherectomy (Self-reported) Hysterectomy-BSO	<55 y ≥55 y <55 y ≥55 y	Ref. 1.00 1.45 (1.07–1.96) 1.55 (1.08–2.23) 1.60 (1.11–2.33) 0.80 (0.19–3.44) 1.31 (0.86–1.98) 1.45 (0.92–2.30) 0.88 (0.38–2.02)	Age, educational attainment, IRSD^d^ score, BMI, endometriosis, fibroids, PCOS^e^	7
Kim et al., 2021 Korea [23]	Nationwide cohort	Population: 671,291 Hysterectomy: 78,961 Follow-up: 12.7 y Incident cases: 12,959 Age: 40.9 ± 10.8	No surgery Hysterectomy without oopherectomy (Hospital record)	<50 y ≥50 y	Ref. 1.00 1.68 (1.58–1.79) 1.66 (1.55–1.78) 1.28 (1.10–1.49)	Age, BMI, smoking, alcohol, indication for surgery, frequency of hospital visit, co-morbidities, history of malignancy, hormone therapy, thyroid disease	9
Guenego et al., 2019 France [24]	Cohort Etude Epidemiologi-que de Femmes de la Mutuelle Générale de l'Education Nationale (E3N)	Population: 89,340 Hysterectomy: 16,064 Incident cases: 412 Follow-up: 9.9 y for cases 21.4 y for non-cases Age hysterectomy: 53.1 ± 6.2 y Age no hysterectomy: 48.8 ± 6.5 y Benign indication	No surgery Any hysterectomy Hysterectomy without oopherectomy (Self-reported) Hysterectomy-BSO	≤40 y 40–45 y >45 y	Ref. 1.00 2.15 (1.72–2.69) 1.96 (1.46–2.65) 2.69 (1.78–4.08) 1.97 (1.32–2.93) 2.08 (1.57–2.74) 2.35 (1.77–3.11)	Age, smoking, dysthyroidism, benign thyroid disease, BMI, age at menarche, use of oral contraceptives, infertility treatment, parity and age at first full-term pregnancy, age at menopause and use of MHT^f^	7
Falconer et al., 2017 Sweden [25]^i^	Nationwide, Population-based cohort	Population: 5,379,882 Hysterectomy: 90,235 Age: 51.1 ± 11.09 No hysterectomy: 5,379,843 Age: 42.4 ± 21.86 y Follow-up: 37 y Incident cases: 2934 Benign indication	No surgery Any hysterectomy (Hospital record)		Ref. 1.00 1.76 (1.45–2.14)	Age at surgery, educational level and parity	9
Luo et al. 2016 USA [26]	Cohort Women's Health Initiative (WHI)	Population: 127,566 Hysterectomy: 46,852 Age: 50–79 y Follow-up: 14,4 y Incident cases: 344	No surgery Any hysterectomy Hysterectomy without oopherectomy (Self-reported) Hysterectomy-BSO	<40 y 40–50 y >50 y	Ref. 1.00 1.46 (1.16–1.85) 1.45 (1.08–1.94) 1.57 (1.13–2.17) 1.50 (1.12–1.99) 1.27 (0.86–1.88) 1.48 (1.13–1.93)	Age, education, smoking, BMI, physical activity, alcohol, thyroid disease	7
Altman et al., 2016 Sweden [27]	Population-based cohort	Population: 5,379,882 Hysterectomy: 111,595 Incident cases: 119 Follow-up: 14,4 Benign indication	No surgery Any hysterectomy Hysterectomy-BSO (Hospital record)		Ref. 1.00 1.76 (1.45–2.14) 1.11 (0.66–1.88)	Age, calendar year, parity, education	9
Braganza et al., 2014 USA [28]	Cohort Prostate, Lung, Colorectal, and Ovarian Cancer Screening Trial	Population: 70,047 Hysterectomy: not reported Age: 50–78 y, median 62y Follow-up: 11 y Incident cases: 127	No surgery Any hysterectomy Hysterectomy-BSO (Self-reported)		Ref. 1.00 1.22 (0.80–1.86) 1.21 (0.71–2.06)	Age, education, race, marital status, family history of thyroid cancer, baseline body mass index, smoking status	9
Kabat et al., 2012 USA [29]	Cohort Women's Health Initiative (WHI)	Population: 145,007 Hysterectomy: not reported Follow-up: 12.7 y Incident cases: 296	No surgery Any hysterectomy (Self-reported)		Ref. 1.00 1.28 (0.99–1.67)	Age, education, height history of goiter/nodules, smoking, alcohol	7
Wong et al., 2006 China [30]	Nested case-cohort	Population: 267,400 Subcohort non-cases: 3187 Hysterectomy: 130 Follow-up: 10 y Incident cases:130	No surgery Any hysterectomy (Self-reported)		Ref. 1.00 0.94 (0.29–3.05)	Age at first live delivery, number of live births, age at first live delivery	7
Truong et al., 2005 New Caledonia [31]	Population-based case-control	Cases: 293 Controls: 354	No surgery Any hysterectomy Hysterectomy without oopherectomy (Self-reported) Hysterectomy-USO Hysterectomy-BSO	<43 43–48 ≥49	Ref. 1.00 1.5 (0.8–2.8) 1.5 (0.7–3.3) 1.8 (0.7–4.8) 1.3 (0.4–3.9) 0.9 (0.3–3.2) 1.6 (0.3–8.7) 1.5 (0.5–4.5)	Age, ethnic group	7
Luoto et al., 2003 Finland [32]	Population-based cohort	93,282 Hysterectomy: 58,721 Follow up: 6 y Incident cases: 118	No surgery Any hysterectomy Hysterectomy-USO Hysterectomy-BSO (Hospital record)		Ref. 1.00 1.52 (1.15–1.96) 0.89 (0.36–1.82) 1.41 (0.96–2.00)	None	7
Rossing et al., 2000 USA [33]	Population-based case-control	Cases: 410 Controls: 574 Hysterectomy: 214 Age: 45–64 Fibroids, dysmenorrhea endometriosis, cervical cancer, prolapsed uterus/other uterine problem	No surgery Any hysterectomy Hysterectomy without oopherectomy (Self-reported) Hysterectomy-BSO	≤30 31–40 >41	Ref. 1.00 1.8 (1.1–3.0) 2.2 (1.3–4.0) 1.8 (0.7–4.9) 2.0 (1.1–3.9) 1.6 (0.8–3.2) 1.3 (0.6–2.6)	Age, county	8
Mack et al., 1999 USA [34]	Individually neighbourhood matched case-control	Cases: 292 Controls: 292 Hysterectomy: 62 Age: 15–54 y	No surgery Any hysterectomy Hysterectomy without oopherectomy (Self-reported) Hysterectomy-USO Hysterectomy-BSO		Ref. 1.00 1.9 (1.0–3.8) 1.0 (0.4–2.4) 2.2 (0.5–9.3) 6.5 (1.1–38.1)	Age, study period	8
Luoto et al., 1997 Finland [35]	Retrospective cohort	Hysterectomy: 25,382 Follow-up: 20.5 y No hysterectomy: ∼25,382 Follow-up: 19.9 y Incident cases: 71	No surgery Any hysterectomy (Self-reported)		Ref. 1.00 2.1 (1.5–3.1)	Age, education, parity, and follow-up	7

a Quality Score; ^b^Body Mass Index; ^c^Socio-Economic Indexes for Areas; ^d^ Index of Relative Socioeconomic Disadvantage; ^e^Polycystic Ovarian Syndrome; ^f^Menopausal Hormone Therapy; ^g^Excluded from meta-analysis because included in Altman et al. [27]

The study-specific quality scores of selected articles are reported in the last right column of Table 1. All our studies can be considered of high-quality since the quality scores ranged from 7 to 9 (median: 7.5; mean: 7.9). In particular, six records had a score of 9 [20,21,23,25,27,28], two of 8 [33,34] and eight of 7 [22,24,26,[29], [30], [31], [32],^35^].

### Meta-analysis

**Fig. 2:**
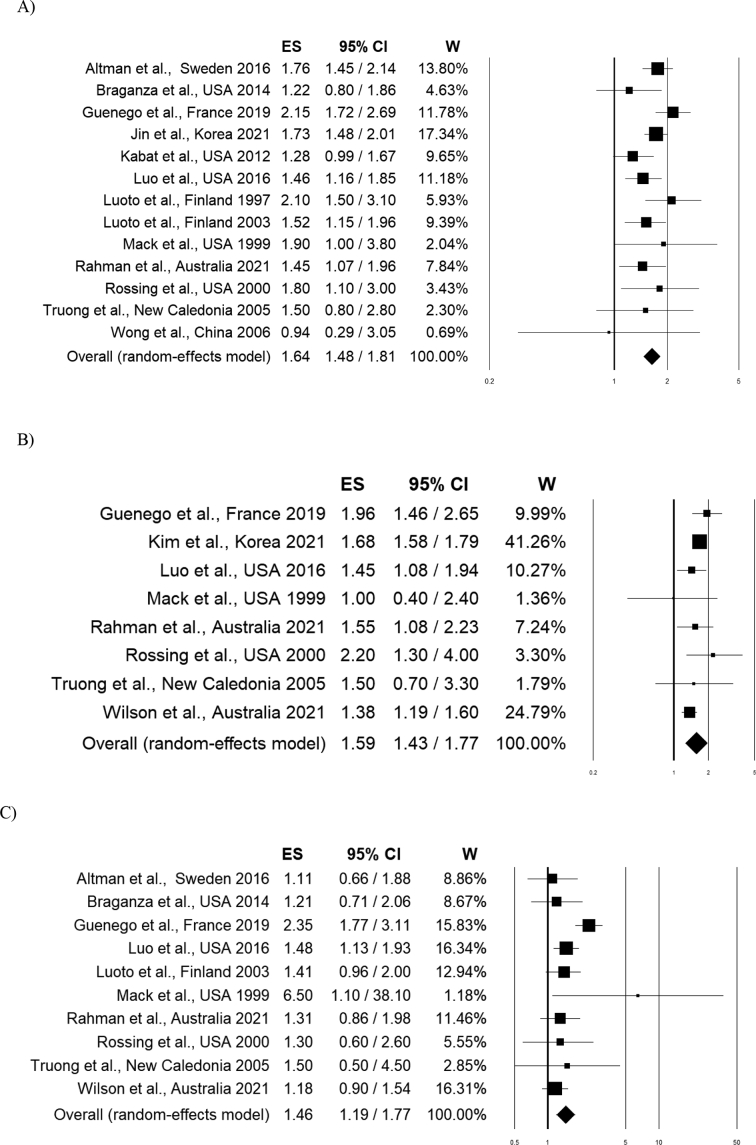
Forest Plot; 2 A) Any type of hysterectomy and thyroid cancer risk; 2B) Any type of hysterectomy without oophorectomy and thyroid cancer risk; 2C) Any type of hysterectomy with bilateral oophorectomy and thyroid cancer risk.

Fifteen out of 16 articles included in the systematic review were used for the overall risk estimation. One study [25] was excluded because it reported the same results, on the same population, of a previous publication [28] carried out by the same authors. Pulling together the data of the 13 studies reporting the risk in association with “any hysterectomy” we found a 64% increment of thyroid cancer risk (OR 1.64, 95% CI 1.48–1.81) (Fig. 2A) (Table 2).

**Table 2:** Results of stratified analysis of the thyroid cancer risk estimates according to the oophorectomy condition.

	Combined risk estimate				Test of heterogeneity		Publication bias	
	N.^b^	Value (95% CI)	p	Q	I^2^%	p	p (Egger test)	p (Begg test)
Any hysterectomy	13	1.64 (1.48–1.81)	<0.0001	16.83	28.68	0.156	0.382	0.542
Self-reported	11	1.62 (1.43–1.84)	<0.0001	16.05	37.69	0.098	0.477	0.697
Hospital record	2	1.67 (1.43–1.96)	<0.0001	0.76	0.00	0.384	–	–
Case-control	4	1.57 (1.25–1.97)	<0.0001	0.88	0.00	0.829	0.268	0.497
Cohort	9	1.64 (1.45–1.86)	<0.0001	15.69	49.01	0.047	0.313	0.404
Hysterectomy without oophorectomy	8	1.59 (1.43–1.77)	<0.0001	10.19	31.32	0.178	0.585	0.621
Self-reported	6	1.65 (1.40–1.95)	<0.0001	4.41	0.00	0.492	0.648	0.573
Hospital record	2	1.54 (1.27–1.87)	<0.0001	5.76	82.64	0.016	–	–
Case-control	4	1.61 (1.23–2.11)	0.001	2.35	0.00	0.504	0.692	0.497
Cohort	4	1.59 (1.38–1.82)	<0.0001	7.83	61.71	0.050	0.694	1.000
Age at surgery <45 y	7	1.81 (1.51–2.17)	<0.0001	6.45	6.93	0.375	0.689	0.652
Age at surgery >45 y	7	1.38 (1.13–1.69)	0.002	13.07	54.1	0.042	0.598	0.453
Hysterectomy with oophorectomy	14	1.39 (1.16–1.67)	<0.0001	22.45	42.1	0.049	0.785	0.352
Case-control	6	1.44 (1.04–1.99)	0.026	3.39	0.00	0.64	0.097	0.015
Cohort	8	1.34 (1.06–1.70)	0.013	19.06	63.27	0.008	0.161	0.216
Hysterectomy-BSO^c^	10	1.46 (1.19–1.77)	<0.0001	17.98	49.94	0.035	0.942	0.531
Case-control	4	1.41 (1.00–1.98)	0.051	3.03	1.12	0.386	0.212	0.042
Cohort	6	1.45 (1.14–1.85)	0.003	14.83	66.28	0.011	0.501	0.573
Hysterectomy-USO^d^	4	0.99 (0.62–1.57)	0.967	1.76	0.00	0.623	0.069	0.174
Case-control	2	1.92 (0.64–5.79)	0.247	0.08	0.00	0.780	–	–
Cohort	2	0.86 (0.52–1.43)	0.561	0.01	0.00	0.914	–	–

aThe risk estimates were calculated using the random-effects model; ^b^Number of data used to calculate the risk; ^c^Bilateral Salpingo Oophorectomy; ^d^Unilateral Salpingo Oophorectomy.

Stratifying the analysis according to the method used for hysterectomy assessment, we observed a cancer risk increment of 62% and 67% for the self-reported and for the hospital record, respectively (Table 2). Stratifying by study design, we observed a cancer risk increment of 57% for the case-control and 64% for the cohort studies with a moderate heterogeneity (I^2^: 49.01) for cohort studies (Table 2).Similarly, when considering the effect of “hysterectomy without oophorectomy” we observed a 59% increment of thyroid cancer risk (OR 1.59, 95% CI 1.43–1.77; I^2^: 31.32) (Fig. 2B) (Table 2).In this case, it was possible to calculate the risk as a function of age at surgery with an increment of 81% and 38% for age < 45 and > 45 years, respectively (Table 2). The pooled analysis of data on hysterectomy with oophorectomy (14 studies) and the risk of thyroid cancer showed association (OR 1.39, 95% CI 1.16–1.67; I^2^: 42.10%, *p* = 0.049) (Table 2). An increment of thyroid cancer risk was also observed when hysterectomy was associated to BSO (OR 1.46, 95% CI 1.19–1.77) with a moderate heterogeneity (I^2^: 49.94) (Fig. 2C) (Table 2). No effect was reported when hysterectomy was associated to USO (OR 0.99, 95% CI 0.62–1.57) (Table 2).

### Sensitivity analysis

Sensitivity analyses investigating the influence of a single study on the thyroid cancer risk estimates suggested that these were not substantially modified by any single study. Indeed, the thyroid cancer risk estimates associated to “any hysterectomy” ranged from 1.60 (95% CI 1.47–1.74, *p* < 0.0001), omitting the study of Guenego et al. [24], to 1.66 (95% CI 1.51–1.83, p < 0.0001), omitting the study of Braganza et al. [28] Similarly, the thyroid cancer risk estimates associated to “hysterectomy without oophorectomy” ranged from 1.53 (95% CI 1.34–1.74, p < 0.0001), omitting the study of Kim et al. [23], to 1.68 (95% CI 1.58–1.78, p < 0.0001), omitting the study of Wilson et al. [21]

### Publication bias

**Fig. 3:**
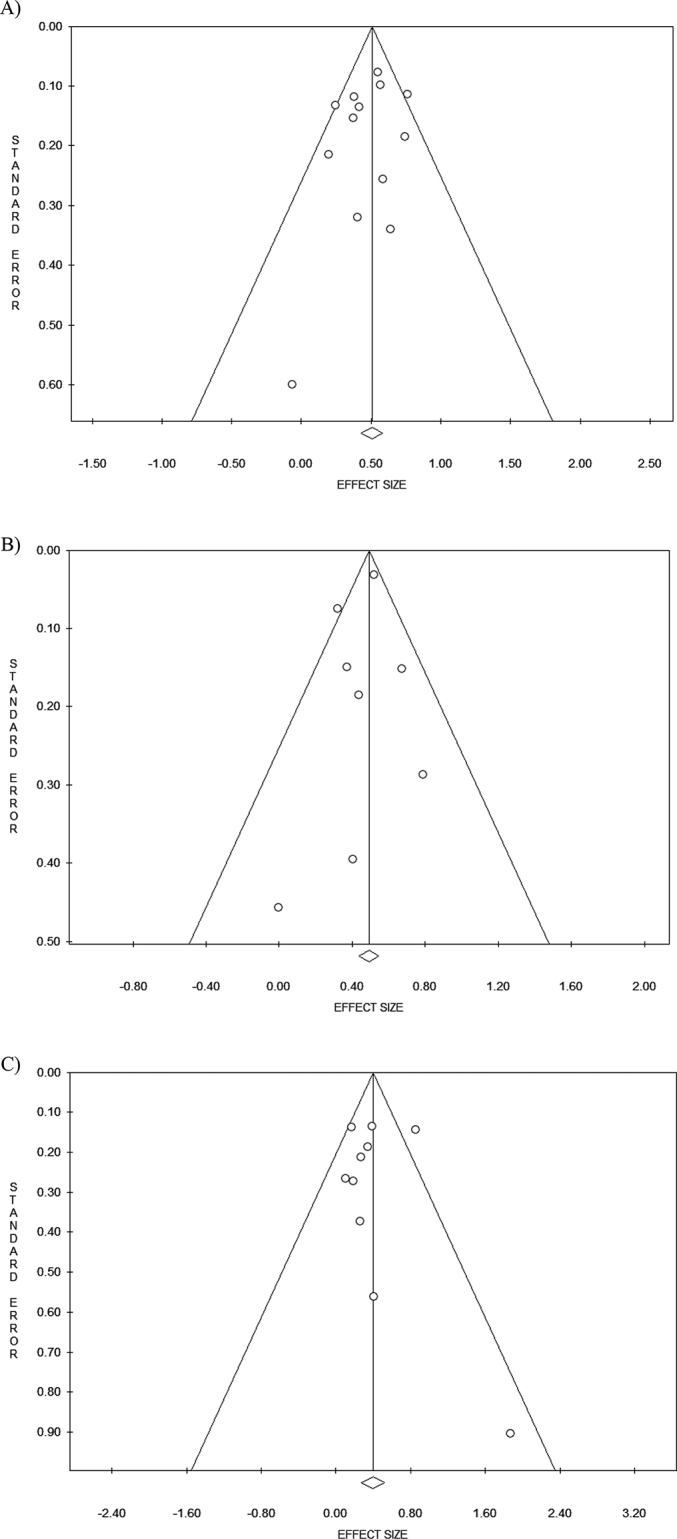
Funnel Plot; 3 A) Any type of hysterectomy and thyroid cancer risk; 3B) Any type of hysterectomy without oophorectomy and thyroid cancer risk; 3C) Any type of hysterectomy with bilateral oophorectomy and thyroid cancer risk.

No significant publication bias was detected with Egger's or Beggs method (Table 2) and analysing the symmetry of funnel plots (Fig. 3).

## Discussion

### Main finding of this study

The incidence of thyroid cancer continues to increase globally,1 presenting a challenge in identifying unestablished risk factors. Current meta-analysis provides a significant update of Caini's study [13]. We found that hysterectomy, with or without oophorectomy, was associated with an elevated relative risk of developing thyroid cancer. Convincing results were obtained both in case-control and cohort studies. Thyroid cancer risk was increased by 64% for women undergoing hysterectomy in comparison to no surgery. Hysterectomy without oophorectomy was a stronger predictor of risk than hysterectomy with oophorectomy (59% vs 39%). Furthermore, hysterectomy associated to BSO increased the risk by 46% while we found no association between hysterectomy associated to USO and thyroid cancer risk. This last finding should be considered with caution as it is obtained from a small number of studies in which partial ovarian removal was considered. We also found association between hysterectomy and risk of thyroid cancer in relation to the age in which the woman underwent hysterectomy: women with hysterectomy without oophorectomy had increased risk of thyroid cancer with decreasing age at surgery. In any case, these data are of particular importance and could help in the decision-making process regarding these surgeries in terms of thyroid cancer risk.

### What is already known on this topic

The biological mechanism underlying the association between hysterectomy and thyroid cancer is unclear, although both hormonal and iatrogenic factors have been suggested. Furthermore, the effect of gynaecological surgery has been shown to be associated to an alteration of lipid peroxidation levels, which may induce DNA damage and promote mutations in proto-oncogenes and tumour suppressor genes [36].

Thyroid cancer is the most common endocrine malignancy and female hormones can contribute to modulate cellular proliferation and cell cycle progression through receptor-mediated transcriptional mechanisms [[37], [38], [39]]; moreover, previous studies reported the expression of progesterone and estrogenic receptors in thyroid cancer in various degrees [40].

Evidence suggests that oestrogens may contribute to gender differences in the immune pathways [41]. and response [42], even though the role of sex hormones in the immunologic escape of cancer remains unclear [43].

Steroid hormones, such as oestrogen, act through their cognate receptors, i.e., oestrogen receptor alfa (ERα) and oestrogen receptor beta (ERβ) [44].

ERs belong to the nuclear receptor superfamily, which act as transcription factors. Oestrogen binding to the nuclear receptors is responsible for a nuclear translocation, with the consequent activation of genomic pathways and the transcription of multiple target genes. ERα promotes DNA transcription, while ERβ inhibits it; ERα plays a role in tumorigenesis by stimulating cell proliferation, while ERβ seems to have a significant antitumor activity [45].

In a study that examined association of endogenous sex hormone levels with hysterectomy, oophorectomy status, age or years since menopause, it was demonstrated that androgen levels were greatly decreased in women who undergo simple hysterectomy in comparison with natural menopause. In addition, testosterone levels were 40% lower in hysterectomized women with bilateral oophorectomy compared to not hysterectomized women, with intermediate levels of testosterone observed in hysterectomized women with ovarian conservation [46].

These results demonstrate that ovaries remain a critical source of androgen throughout the lifespan of a woman. Since both hysterectomy with oophorectomy and hysterectomy without oophorectomy have the potential to induce evident changes in hormone levels, they can also influence the risk of hormones related cancers. Further studies are needed to evaluate the effects of hormone replacement after oophorectomy.

The adverse association between hysterectomy and thyroid cancer may be a consequence of the underling conditions leading up to hysterectomy. The most common diagnosis associated with hysterectomy are uterine fibroids, abnormal uterine bleeding, prolapsed, endocrine disorders and endometriosis [47]. Indeed, it has been demonstrated that endometriosis was associated with a higher risk of thyroid cancer [48].

The carcinogenic potential of conditions that lead to a hysterectomy is poorly understood, but hypothesized to be multidimensional in aetiology, involving hormonal, genetic and immunological factors [49].

Finally, it is necessary to consider that women undergoing hysterectomy are more medicalized and therefore more in touch with the health care system and more concerned about their own health, so they may have a higher likelihood of being diagnosed with thyroid cancer than the general population.

### Strengths and limitations

Sensitivity and publication bias analyses revealed that our results are stable. Moreover, in our systematic review have been included many cohort studies, of good quality, that reinforce our results. It is important to notice that the number of people that have been considered by our systematic review is 7.575.968 and that the meta-analysis showed a consistently results of 64% increment of thyroid cancer risk in association with any type of hysterectomy.

The principal weakness of our study, instead, is that, even if a high agreement was registered between self-reported hysterectomy data (i.e. year of surgery) and hospital records in studies that validated exposure information, for several studies the hysterectomy status was not verified with medical records and it is self-reported only [[49], [50], [51], [52]]. Actually, self-reported hysterectomy history is a reliable data, instead it is necessary to pay attention to the interpretation of self-reported oophorectomy history. An incorrect self-reported oophorectomy status could lead to a misclassification error of predictor groups, which may invalidate results. Moreover, most of women in our studies were Caucasians, so our conclusions may not be applicable to other ethnic groups.

## Conclusions

Oophorectomy or hysterectomy should be proposed to women who have a high-penetrance susceptibility genes for ovarian cancer and/or endometrial cancer, because of the high life-time risk of developing these types of cancers [53], while women who are not at high risk of ovarian cancer or endometrial cancer, the gynaecological surgery should be a careful choice, because of the increasing risk of thyroid cancer, as find in our study, and of colorectal and kidney cancer's risk, as reported in two previous meta-analysis [10,11].

Our review was able to account risk factors for thyroid cancer, however, other confounding factors should be taken in consideration, such as indications for hysterectomy, type of hysterectomy (mini-invasive as laparoscopic, vaginal, or robotic and invasive as abdominal hysterectomy) and an eventual substitutive hormone therapy given in case of ovariectomy, which can influence woman cancer history. Despite challenges, additional studies are needed to determine the biological mechanisms of these associations and cohort studies with prolonged follow-up are essential to verify our findings.

In conclusion, our findings from this meta-analysis suggest that women undergoing hysterectomy have an increased relative risk of thyroid cancer. This relative risk depends on frequency of hysterectomy and thyroid cancer, however, the proportion of thyroid cancers attributable to hysterectomy may be substantial, given that approximately 45% of women are estimated to undergo this procedure by the age of 70 years.

## Author contribution

all authors have contributed significantly. F.R. provided the idea and was responsible for the study design. Authors C.M. and F.R. performed the review and C.M. and G. I. wrote the article. Author F.R. and R.P. analysed the data, edited pictures and performed the statistical analysis. C.M and G.I. performed reference collection. All authors revised the manuscript and approved the final version. All authors are included in the author list and are aware that the manuscript was submitted.

## Funding/Support

The authors received no financial support for this research.

## Declaration of Competing Interest

The authors declare that they have no known competing financial interests or personal relationships that could have appeared to influence the work reported in this paper.

## Appendix A Supplementary data

Supplementary material: studies' quality evaluation Image 1
